# Supplementary material for: Navigation and the efficiency of spatial coding: insights from closed-loop simulations
Source: Brain Struct Funct. 2023 Apr 8;229(3):577–92. doi: 10.1007/s00429-023-02637-8 (PMC10978723; doi:10.1007/s00429-023-02637-8)
Supplement: Supplementary file 1 — Supplementary file1 (DOCX 1323 kb) [file 429_2023_2637_MOESM1_ESM.docx]

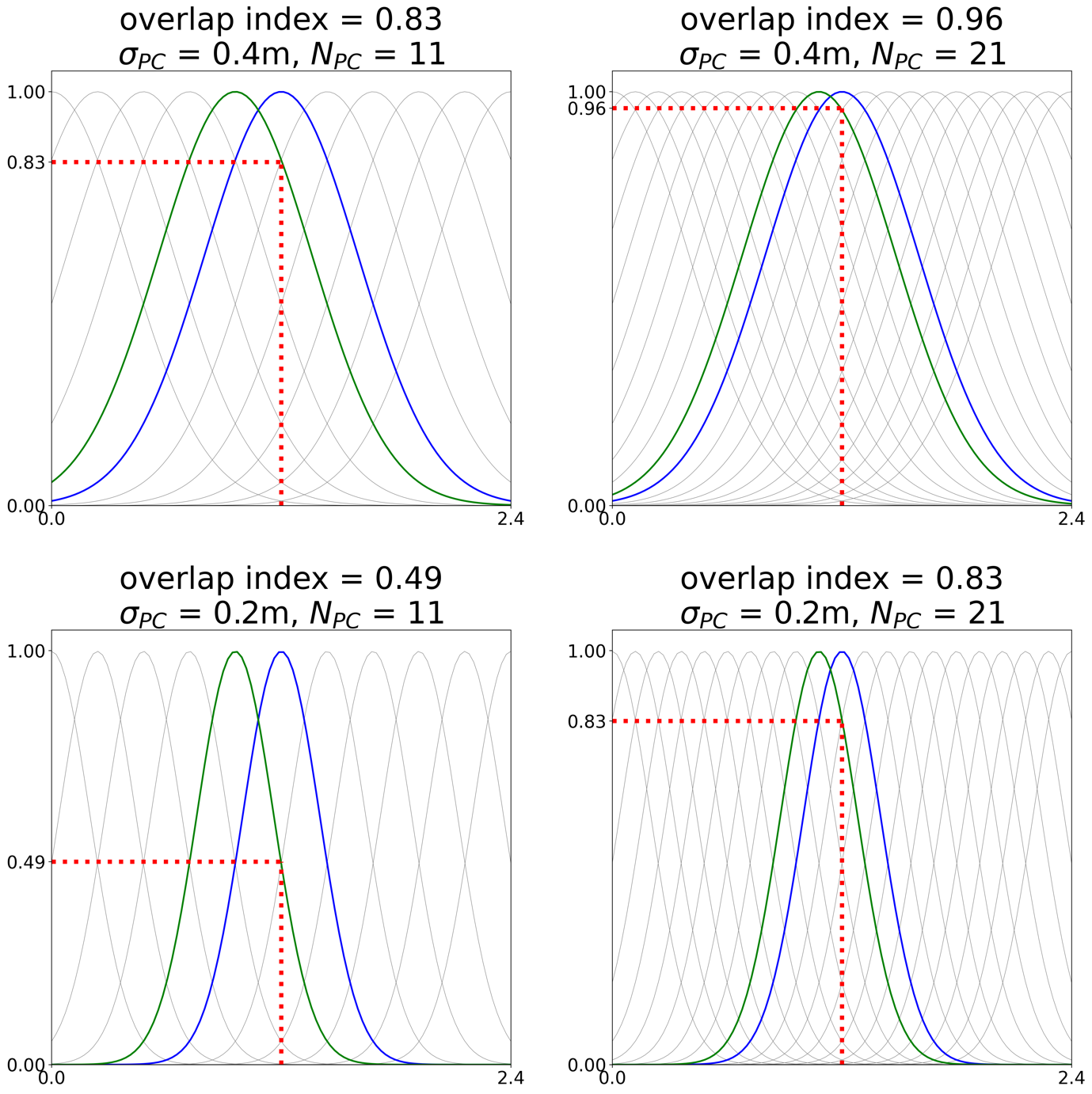


Fig. A1 Illustration of the overlap index in 1-d. Bottom-Left: tuning curves of *N_PC_* = 11 place cells with field size *σ*_PC_ = 0.2, homogeneously distributed over one edge of the environment (2.4 m). The overlap index is defined as the relative firing rate of the nearest neighboring place cell (green) in the center of a given place cell (blue). In this example, overlap index= 0.49. The overlap index increases with the cell number (left to right) and with the place field size (bottom to top). Since both cases scale in the same way (overlap index= 0.83), one can maintain a constant overlap, if decreasing the cell number and increasing field size (or vice a versa) by the same factor, i.e., such that *N_PC_σ*_PC_= const


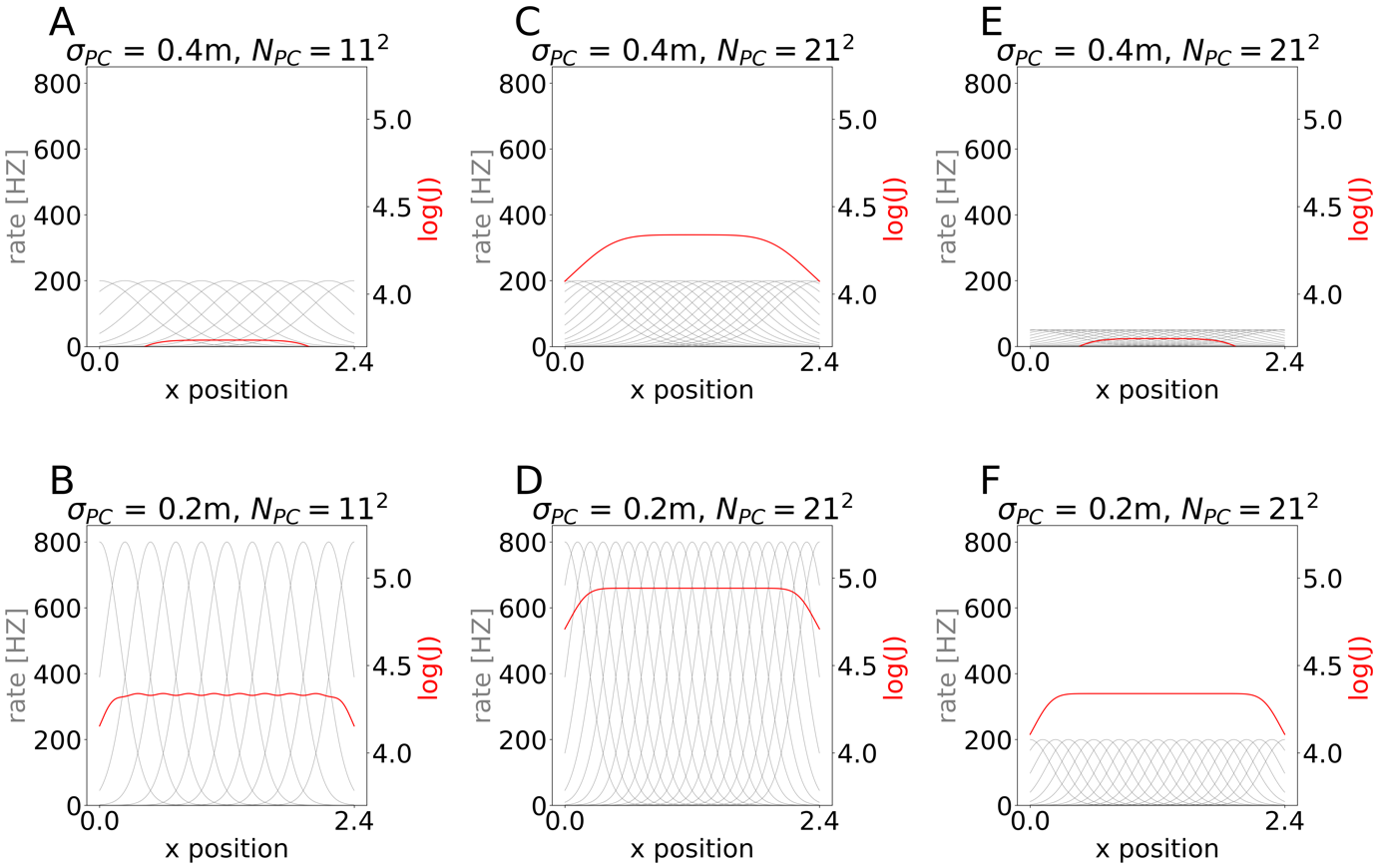


Fig. A2 Illustration of Fisher information. Binary logarithm of Fisher information for six place cell populations characterized by different values of cell number, field size, and maximum individual firing rate. Generally, larger field sizes leads to lower Fisher information (comparing top row to bottom row) and increasing the number of cells increases the Fisher information (comparing 2nd column to 1st). However, in our simulations we keep the summed network activity in the center roughly constant (~3500 Hz) by decreasing the individual maximum firing rate *η*_PC_. This scaling reduces the Fisher information (comparing 3rd column to 2nd). The effects of increasing cell number and decreasing maximum firing rate balance each other (comparing 1st column to 3rd). As a result, scaling cell number in our simulations does not influence the Fisher information under normal circumstances


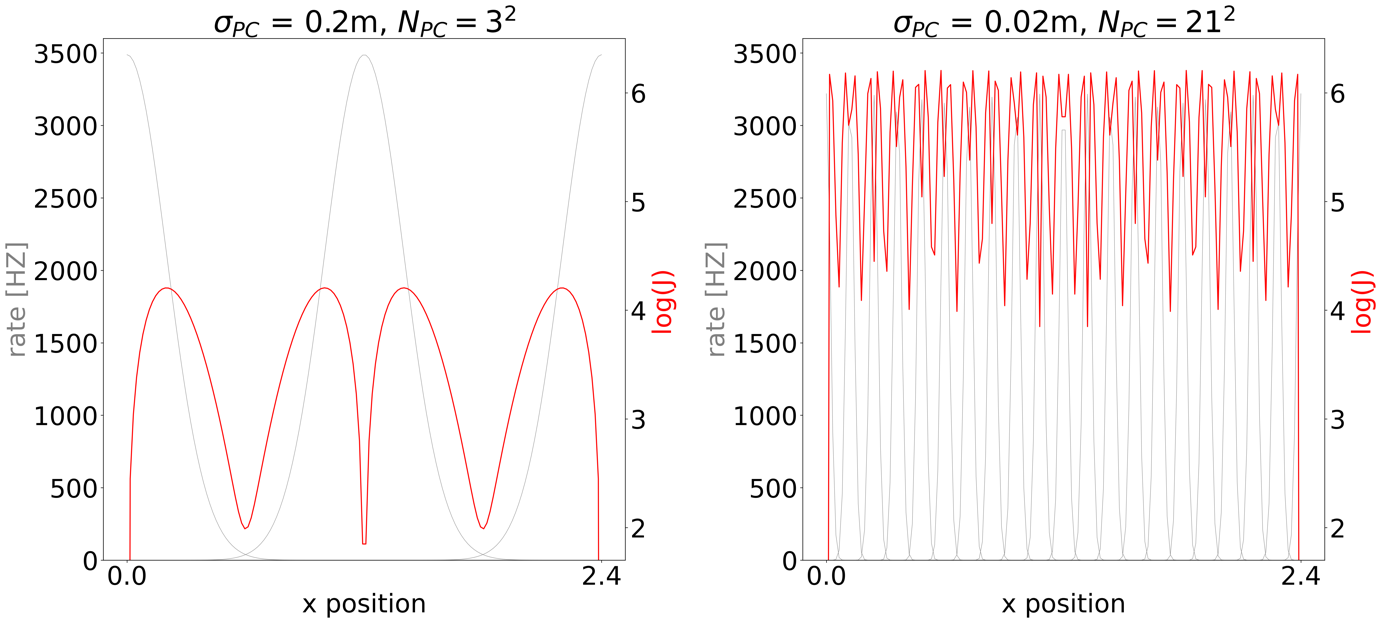


Fig. A3 Fisher information fluctuates greatly when place fields are sparse. The Fisher information for most values of the place cell parameters is fairly homogeneous across the environment. However, that is not the case for very low cell numbers or very small field sizes. Therefore, we chose the minimum Fisher information along the path from the start point to the goal's center to quantify spatial coding by the place cell population, because the locations where Fisher information is low are the bottlenecks of spatial learning. This figure illustrates two examples of extremely heterogeneous Fisher information


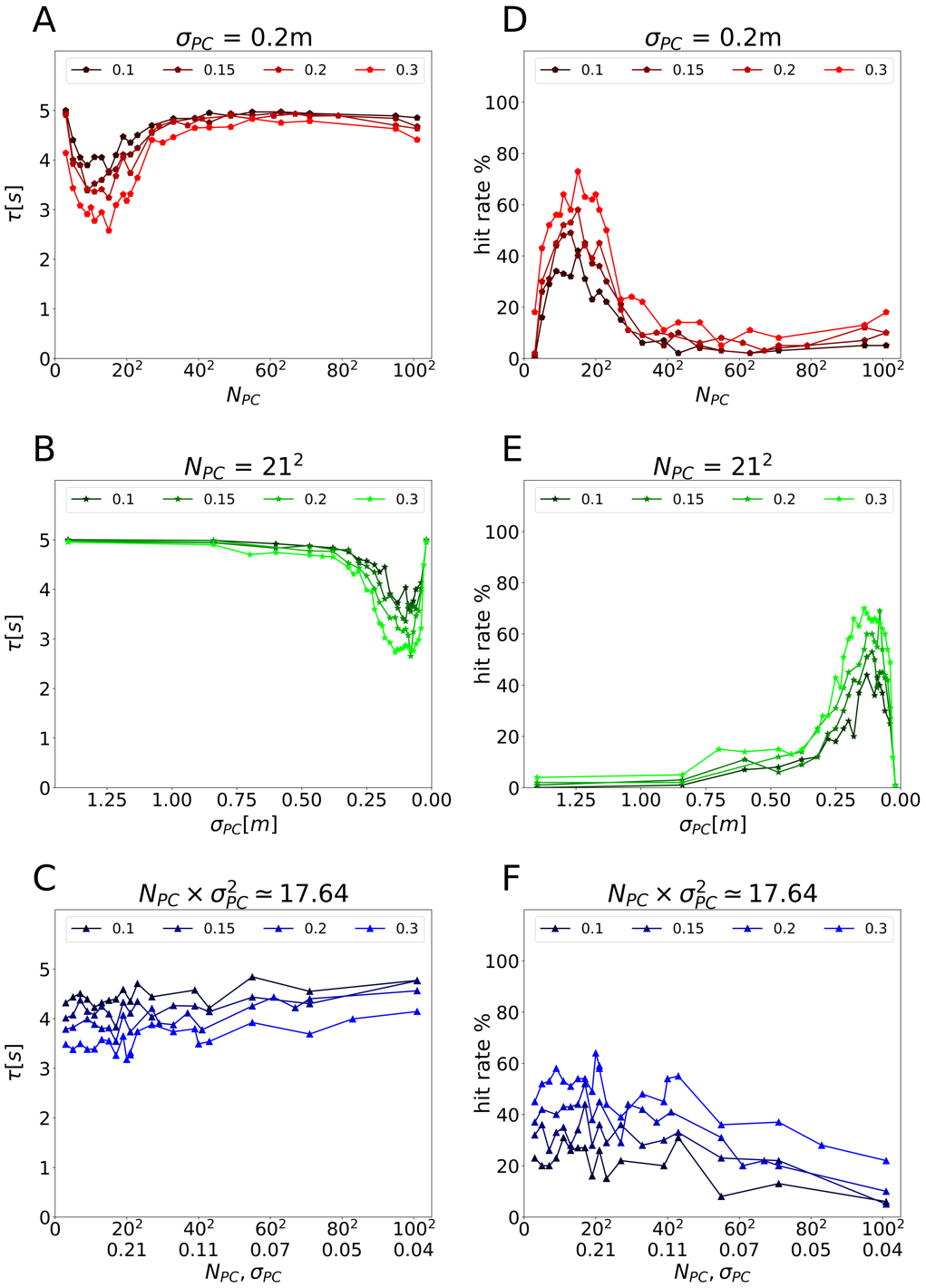


Fig. A4 Navigation performance before learning based on randomly initialized network. Escape latency as a function **A**: of place cell number *N*_PC_ for fixed *σ*_PC_ = 0.2 m, **B**: of place field size *σ*_PC_ for fixed *N*_PC_ = 21^2^, and **C**: of both variables scaled simultaneous such that *N*_PC_ × $\sigma_{PC}^{2}$ = 17.46 m^2^. Each point represents the average trial duration over 100 repetitions. The color represents different goal radii (as indicated). **D**–**F**: The hit rate, i.e. the fraction of trials in which the agent successfully navigates to the goal, shown using the same plotting convention as in A-C
